# Temporal Prognostic Factors in Elderly Patients with Acute Heart Failure: A Cohort Study from a Spanish Emergency Department

**DOI:** 10.3390/geriatrics11010021

**Published:** 2026-02-18

**Authors:** Itziar Ostolaza Tazón, Héctor Alonso Valle, Pedro Muñoz Cacho

**Affiliations:** 1 Department of Emergency, Marqués de Valdecilla University Hospital-Valdecilla Research Institute (IDIVAL), 39008 Santander, Spain; hector.alonso@scsalud.es; 2 Teaching Department of Primary Care Management, Cantabrian Health Service, IDIVAL, 39011 Santander, Spain; pedro.munoz@scsalud.es

**Keywords:** acute heart failure, comorbidity, mortality, prognosis, risk factors

## Abstract

**Background/Objectives:** Acute heart failure (AHF) is a common cause of hospitalization in older adults, associated with high morbidity and mortality. In this population, frailty, comorbidity, and functional variability significantly influence prognosis. This study evaluated short-term (30-day) and long-term (1-year) mortality predictors in elderly patients with AHF treated in the emergency department (HED), considering clinical variables, comorbidities, and precipitating factors (PFs). **Materials and Methods:** An observational cohort study was conducted based on a secondary analysis of older patients with AHF included in the Epidemiology of Acute Heart Failure in Emergency Departments (EAHFE) registry, treated at Hospital Universitario Marqués de Valdecilla (HUMV) between 2007 and 2022. Clinical, laboratory, and PF-related variables were collected. The primary outcome was all-cause mortality at 30 days and 1 year. Univariate and multivariate logistic regression analyses were performed. **Results:** A total of 548 patients were included (mean age: 80.7 years), of whom 78.6% required hospitalization, mainly in the Internal Medicine department. Mortality was 11.1% at 30 days and 29.9% at 1 year. Age, valvular heart disease, dementia, and elevated creatinine levels were independently associated with higher mortality. Hypoxemia and low-output symptoms were linked to short-term mortality, while NYHA class III and anemia were associated with long-term mortality. Among PFs, acute coronary syndrome (ACS) was related to worse short-term outcomes, whereas rapid atrial fibrillation (AF) was inversely associated with long-term mortality. **Conclusions:** The prognostic relevance of risk factors differs between short- and long-term outcomes in older patients with AHF. Incorporating clinical characteristics and PFs into risk stratification models may support individualized management and guide follow-up strategies tailored to the geriatric profile. This multidimensional approach is essential to improve clinical decision-making and outcomes in a highly vulnerable population.

## 1. Introduction

Acute heart failure (AHF) is a clinical syndrome defined by the sudden onset or rapid worsening of heart failure (HF) symptoms that require urgent medical intervention [1]. It is a major cause of morbidity, mortality, and healthcare resource utilization worldwide, particularly among older adults, due to its high prevalence and frequent hospital admissions and readmissions.

The progressive aging of the population, together with improved survival from other cardiovascular diseases, has led to a sustained increase in the number of elderly patients with HF. The prevalence of HF doubles with each decade of life, from approximately 1% in individuals younger than 55 years to more than 10% in those over 70, and nearly 20% in those older than 80 years [2,3,4,5,6]. By 2050, it is estimated that 17% of the global population will be older than 85 years [7,8,9], anticipating a substantial rise in hospitalizations, demand for healthcare resources, and clinical complexity.

In older patients, HF is characterized by a distinct clinical and prognostic profile. It frequently coexists with comorbidities such as diabetes mellitus (DM), atrial fibrillation (AF), and chronic kidney disease, which influence disease progression and therapeutic response [10,11,12,13,14,15]. Geriatric syndromes including frailty, sarcopenia, and cognitive decline further reduce physiological reserve and increase vulnerability to acute decompensation [4,16]. Polypharmacy, highly prevalent in this population, adds to the risk of drug interactions, adverse events, and poor adherence, complicating the application of guideline-directed pharmacological therapy [17,18].

These factors highlight the need for an individualized and multidisciplinary approach to HF management in the elderly, combining optimization of medical treatment with functional and cognitive assessment, and prioritizing quality of life and prevention of readmissions over strategies exclusively focused on survival.

Despite the predominance of older patients among those with HF, most available evidence derives from studies conducted in younger populations with fewer comorbidities. As a result, current therapeutic recommendations and prognostic models may not be fully applicable to elderly patients, in whom treatment goals must balance clinical efficacy with functionality and quality of life. This lack of representativeness limits the extrapolation of classical study results to clinical practice, particularly in the HED setting, where many episodes of acute decompensation are managed.

HEDs are the usual point of entry for elderly patients with AHF [19]. Following initial evaluation and stabilization, most require hospital admission, mainly to Internal Medicine units [20,21,22]. However, a considerable proportion experience early readmissions or die within the first weeks after discharge, underscoring the need for effective risk stratification tools from the earliest stages of care. Traditionally, risk stratification has relied on clinical and laboratory parameters [23,24,25]. More recently, other determinants such as precipitating factors (PFs) have emerged, although they have received less attention despite their prognostic relevance [26,27].

The aim of the present study is to identify the main prognostic determinants of mortality in elderly patients treated for AHF in a HED, assessing their impact both in the short term (30 days) and in the long term (1 year). Given that the clinical course of HF in older adults may vary substantially depending on the stage of the disease, it is essential to understand which variables differentially influence early versus late outcomes.

This temporal approach aims to distinguish the clinical determinants that shape prognosis across different phases of the disease, thereby supporting more accurate risk stratification and individualized therapeutic planning. Ultimately, the goal is to optimize initial emergency care and enhance continuity of care by identifying those factors that warrant closer monitoring and follow-up in this highly vulnerable patient population.

## 2. Materials and Methods

### 2.1. Study Design and Setting

This study is based on a secondary analysis of the EAHFE (Epidemiology of Acute Heart Failure in Emergency Departments) registry, an observational, multicenter, multipurpose, non-interventional analytical registry with prospective follow-up in 45 hospitals across Spain. For this analysis, only patients treated at the HED of the Marqués de Valdecilla University Hospital (HUMV) in Cantabria were included.

The design and recruitment process of the EAHFE registry have been described previously [28,29,30]. In brief, the registry prospectively included all consecutive patients who presented to the ED with a clinical syndrome of HF established according to Framingham criteria whose episode met the ESC definition of AHF [1] and who provided written informed consent. Exclusion criteria included inability to complete follow-up, a final hospital discharge diagnosis unrelated to AHF, and AHF occurring in the context of a concurrent ST-segment elevation myocardial infarction (STEMI). The diagnosis was confirmed through review of the medical record and complementary test results by the principal investigator.

In accordance with the national protocol of the EAHFE registry, data collection was conducted prospectively following a standardized design based on short, predefined recruitment periods at the national level. These periods corresponded to the years 2007, 2009, 2011, 2014, 2016, 2018, and 2022. This methodology, established by the EAHFE project itself, ensured methodological consistency, temporal diversity, and representativeness of the cohort of patients with AHF [28,29,30].

### 2.2. Participants

**Figure 1:**
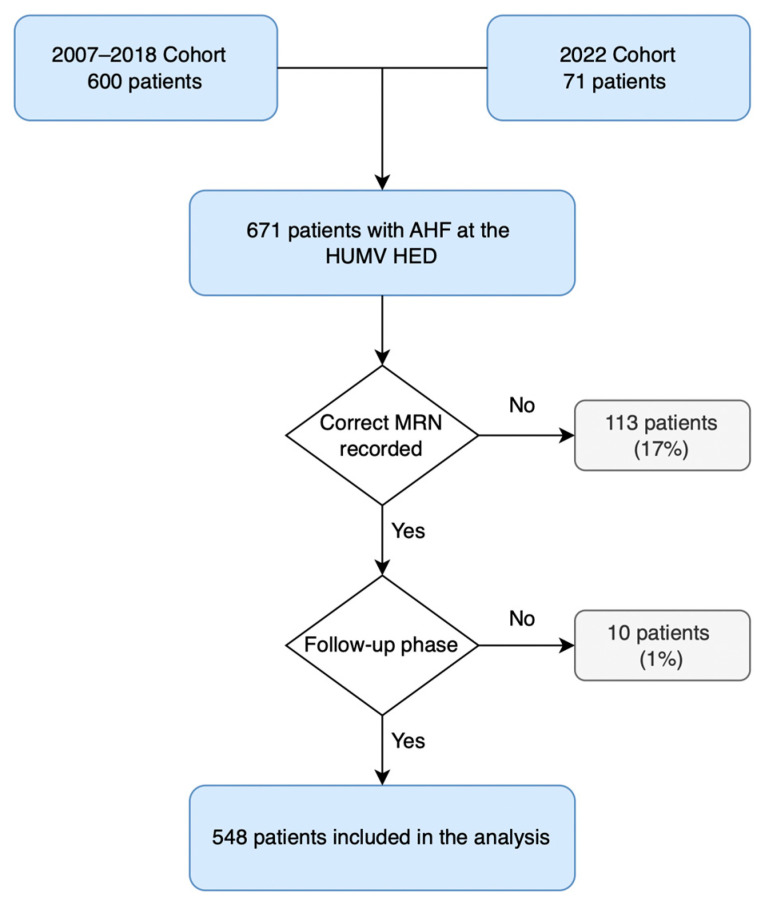
Flowchart of the study cohort selection process (AHF: acute heart failure; HED: hospital emergency department; HUMV: Marqués de Valdecilla University Hospital; MRN: Medical Record Number).

A total of 671 patients with AHF were registered, of whom 123 (18%) were excluded due to inability to complete follow-up, mainly because of missing medical record numbers (MRN) from the 2007 data collection period or residence outside the autonomous region. The final analyzed sample included 548 elderly patients with AHF. The flowchart detailing inclusion and exclusion criteria is presented in Figure 1.

### 2.3. Study Variables

As this was a secondary analysis of the EAHFE registry [28,29,30], the variables included corresponded to those collected prospectively and predefined in the original design of the registry.

A total of 34 independent variables were collected and grouped into different categories: two demographic variables (age and sex); eight comorbidities (DM, ischemic heart disease, AF, atrial flutter, valvular heart disease, chronic obstructive pulmonary disease [COPD], dementia and prior HF); one baseline functional status variable (New York Heart Association [NYHA] functional class); eight PFs for AHF (infection, rapid AF [defined as AF with heart rate ≥ 120 beats/min], rapid atrial flutter [defined as atrial flutter with heart rate ≥ 120 beats/min], anemia, hypertensive crisis, acute coronary syndrome (ACS), specifically non-ST-segment elevation ACS and other ischaemic triggers without ST elevation, treatment non-adherence, and unknow); six clinical variables at admission (respiratory rate, oxygen saturation, AHF type [hypertensive AHF, normotensive AHF, hypotensive AHF without shock, hypotensive AHF with shock, and AHF associated with ACS], low-output symptoms, lower-limb edema and pulmonary crackles); five laboratory variables (hemoglobin, creatinine, potassium, N-terminal pro-B-type natriuretic peptide [NT-proBNP], and troponin); one electrocardiographic (ECG) variable (left ventricular hypertrophy); and three ED management variables (use of intravenous nitroglycerin, use of non-invasive ventilation, and hospital admission).

The primary outcome variable was all-cause mortality at 30 days and 12 months, using the date of HED presentation as the index event.

### 2.4. Statistical Analysis

Categorical variables were described as absolute and relative frequencies, and continuous variables as mean and standard deviation, or median and interquartile range (IQR), according to their distribution (assessed using the Kolmogorov–Smirnov test).

Group comparisons were performed using the Chi-square test or Fisher’s exact test for categorical variables, and Student's *t*-test or Mann–Whitney U test for continuous variables, as appropriate. A *p* value < 0.05 was considered statistically significant.

To identify factors associated with mortality, logistic regression analysis was used. Univariate and multivariate analyses were conducted independently for 30-day and 12-month mortality. A univariate analysis was performed to calculate odds ratios (ORs) with 95% confidence intervals (CIs) using the Wald statistic. Variables with a *p*-value > 0.25 in the univariate analysis were subsequently included in the multivariate analysis, following the criterion proposed by Hosmer and Lemeshow [31], and supported by other reference authors [32]. For model selection, an automatic variable-selection procedure using the backward method was applied. In addition, several models were generated based on current knowledge and pathophysiological rationale and compared with the initial models. For each follow-up period (30 days and 12 months), two multivariable models were constructed using different variable-selection strategies: one derived using the backward conditional method and an alternative model built using the enter method.

To assess the predictive capacity of the different models, the area under the curve (AUC) was used.

Analyses were performed with SPSS version 25.0 (IBM Corp., Armonk, NY, USA) and MedCalc^®^ v23.13 (MedCalc Software Ltd., Ostend, Belgium).

### 2.5. Ethical Considerations

The study was conducted in accordance with the principles of the Declaration of Helsinki (1975) and was revised in 2013 with current regulations on personal data protection. All patients provided written informed consent to participate in the EAHFE registry and to be contacted for follow-up.

The study protocol was approved by the Research Ethics Committee of the Principality of Asturias on 12 January 2022 (project code: CEImPA No. 2022.015). This approval covers the continued use of data collected during previous phases of the EAHFE registry. In addition, the Ethics Committee of Cantabria (IDIVAL) issued a local ratification on 28 January 2022.

## 3. Results

### 3.1. Clinical Profile of the Cohort

A total of 548 patients with a diagnosis of AHF were analyzed. The mean age was 80.7 years, and 49.6% were female. Most patients had multiple comorbidities, the most frequent being AF (35.8%), and ischemic heart disease (23.7%). Prior HF was present in 62.6% of cases, predominantly NYHA functional class I–II (67.70%). The sociodemographic and clinical characteristics of the patients included in this study have been previously described in detail by Ostolaza-Tazón et al. [33] and are summarized in Table 1.

On arrival, patients presented with a mean oxygen saturation of 93.6%, and 6.8% had a respiratory rate exceeding 25 breaths/min. Congestive signs were common (78.1%), with the most frequent being peripheral edema (65.3%) and pulmonary crackles (67.0%). PFs were identified in 69.2% of cases, the most common of which were infection (34.7%) and rapid AF (17.3%). Mean hemoglobin was 12.4 g/dL, and mean creatinine was 1.4 mg/dL. Some of these clinical outcomes were also reported by Ostolaza-Tazón et al. [33] and are reorganized here for the current analysis, as summarized in Table 2 and Table S1.

### 3.2. In-Hospital Course and Mortality

Most patients evaluated in the HED for AHF required hospitalization (76.8%), mainly in Internal Medicine (44.3%), followed by Cardiology (19.9%) and the Short-Stay Unit (6.0%). A small percentage were admitted to Intensive or Coronary Care Units (1.9%) or to other services, such as Geriatrics (6.5%). Conversely, 21.4% of patients did not require admission and were discharged directly from the HED after achieving clinical stability, with outpatient follow-up arranged through their usual primary care or hospital physicians.

During follow-up, 11.1% of patients died within 30 days (61 patients), and the mortality rate increased to 29.9% at 1 year (164 patients) after the index episode. Baseline characteristics stratified by survival status at 30 days and 12 months are presented in Supplementary Table S2.

### 3.3. Prognostic Factors for Mortality

Analysis of prognostic factors revealed significant associations between several clinical variables and mortality across different follow-up periods.

#### 3.3.1. Univariate Analysis of 30-Day Mortality

In the univariate analysis of 30-day mortality, several factors were identified as being associated with an increased risk of death. Among them, each additional year of age (OR 1.08; 95% CI 1.04–1.12; *p* < 0.01), ischemic heart disease (OR 2.12; 95% CI 1.17–3.84; *p* = 0.01), valvular heart disease (OR 2.10; 95% CI 1.13–3.90; *p* = 0.02), and baseline NYHA class IV (OR 11.76; 95% CI 3.47–39.83; *p* < 0.01) stood out. Regarding the AHF episode, both the hypotensive form without shock (OR 6.58; 95% CI 1.81–23.89; *p* < 0.01) and the hypotensive form with cardiogenic shock (OR 14.33; 95% CI 1.84–111.44; *p* = 0.01), as well as episodes precipitated by ACS (OR 28.67; 95% CI 5.28–155.62; *p* < 0.01), were significantly associated with short-term mortality. Creatinine levels (OR 1.39; 95% CI 1.16–1.67; *p* < 0.01) were associated with increased mortality, whereas higher oxygen saturation was associated with lower mortality (OR 0.94; 95% CI 0.91–0.98; *p* < 0.01).

Some variables showed a trend toward association without reaching statistical significance, such as previous AHF episodes (OR 1.58; 95% CI 0.85–2.95; *p* = 0.15), respiratory rate >30 bpm (OR 2.08; 95% CI 0.65–6.61; *p* = 0.22), or hospital admission (OR 2.10; 95% CI 0.93–4.78; *p* = 0.08).

Other factors showed a trend toward a protective effect, although without statistical significance, such as a history of AF (OR 0.69; 95% CI 0.37–1.28; *p* = 0.23), AF as a PF (OR 0.58; 95% CI 0.24–1.40; *p* = 0.23), higher hemoglobin levels (OR 0.91; 95% CI 0.79–1.05; *p* = 0.18), and the presence of left ventricular hypertrophy on ECG (OR 0.30; 95% CI 0.04–2.23; *p* = 0.24).

Other comorbidities and clinical or analytical findings were not associated with short-term mortality, including DM (OR 1.35; 95% CI 0.76–2.39; *p* = 0.31) and COPD (OR 0.69; 95% CI 0.27–1.80; *p* = 0.44). Similarly, symptoms such as lower limb edema (OR 1.04; 95% CI 0.57–1.88; *p* = 0.91) or pulmonary crackles (OR 0.96; 95% CI 0.53–1.74; *p* = 0.88) did not reach statistical significance, nor did other potential PFs of the AHF episode, such as therapeutic non-adherence (not estimable) or hypertensive crisis (OR 0.54; 95% CI 0.07–4.15; *p* = 0.55).

#### 3.3.2. Univariate Analysis of 12-Month Mortality

In the univariate analysis at 12 months, advanced age (OR 1.05; 95% CI 1.03–3.48; *p* < 0.01), valvular heart disease (OR 1.66; 95% CI 1.07–2.57; *p* = 0.02), and creatinine levels (OR 1.26; 95% CI 1.07–1.49; *p* < 0.01) remained significantly associated with mortality. Regarding NYHA functional classes, in addition to class IV, class III (OR 2.88; 95% CI 1.68–4.97; *p* < 0.01) reached statistical significance in long-term prediction, while class II (OR 1.70; 95% CI 1.01–2.88; *p* = 0.05) showed a trend toward significance without fully reaching it.

Additionally, new predictors emerged that had not reached statistical significance at 30 days, such as a history of previous AHF episodes (OR 2.05; 95% CI 1.37–3.07; *p* < 0.01) and hospital admission (OR 2.14; 95% CI 1.31–3.48; *p* < 0.01). Rapid AF as a PF (OR 0.57; 95% CI 0.34–0.97; *p* = 0.04), the presence of left ventricular hypertrophy on ECG (OR 0.33; 95% CI 0.11–0.96; *p* = 0.04), and hemoglobin levels (OR 0.85; 95% CI 0.77–0.92; *p* < 0.01) also reached statistical significance and were associated with lower mortality.

Other factors that were significant at 30 days, such as ischemic heart disease (OR 1.46; 95% CI 0.96–2.21; *p* = 0.08), ACS as a PF (OR 2.01; 95% CI 0.97–4.20; *p* = 0.06), or the hypotensive form with cardiogenic shock (OR 3.45; 95% CI 0.65–18.44; *p* = 0.15), showed a similar trend but did not reach significance at 12 months. Likewise, oxygen saturation, which had shown short-term association, lost significance at 12 months (OR 0.99; 95% CI 0.95–1.02; *p* = 0.39).

Potassium levels (OR 1.25; 95% CI 0.98–1.58; *p* = 0.07), which had shown a trend toward worse prognosis at 30 days, continued to show that trend without reaching statistical significance. Similarly, DM (OR 1.47; 95% CI 1.01–2.14; *p* = 0.05) showed a trend toward increased long-term mortality, without reaching statistical significance.

Other clinical findings such as lower limb edema (OR 1.12; 95% CI 0.76–1.65; *p* = 0.56) or pulmonary crackles (OR 0.97; 95% CI 0.66–1.43; *p* = 0.87), as well as most PFs (infection, anemia, hypertensive crisis, therapeutic non-adherence, or unknown cause), were not associated with long-term mortality.

Supplementary Tables S3 and S4 present the complete results of the univariate analyses corresponding to the 30-day and 12-month follow-up periods, respectively.

### 3.4. Multivariable Analysis of Prognostic Factors

Multivariable analysis confirmed similar associations to those observed in the univariate analysis.

#### 3.4.1. Multivariate Analysis of 30-Day Mortality

**Figure 2:**
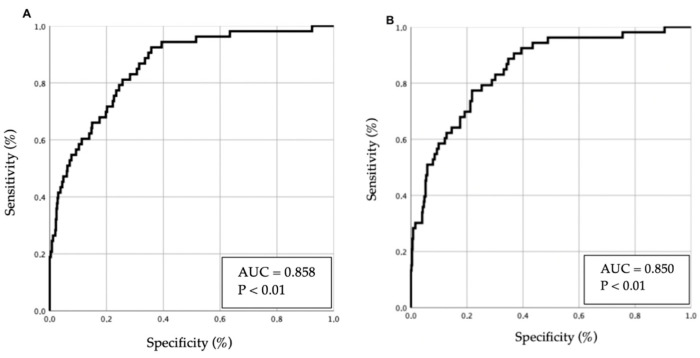
ROC curves of multivariable models for predicting 30-day mortality in patients with acute heart failure. Panels (**A**,**B**) correspond to model 1 and model 2, respectively. The diagonal line represents chance (AUC = 0.5); ROC curves illustrate each model’s discriminative ability.

Two multivariable models were developed using different variable-selection methods (conditional backward method and enter method), and their results are presented in Table 3. Predictive performance is illustrated in Figure 2 (receiver operating characteristic [ROC] curves).

In the multivariate analysis of 30-day mortality, age, ischemic heart disease, valvular heart disease, and hypotensive forms of AHF (with or without cardiogenic shock) remained significant predictors. Among comorbidities, previous episodes of AHF showed a trend toward significance, although without reaching statistical relevance.

#### 3.4.2. Multivariate Analysis of 12-Month Mortality

**Figure 3:**
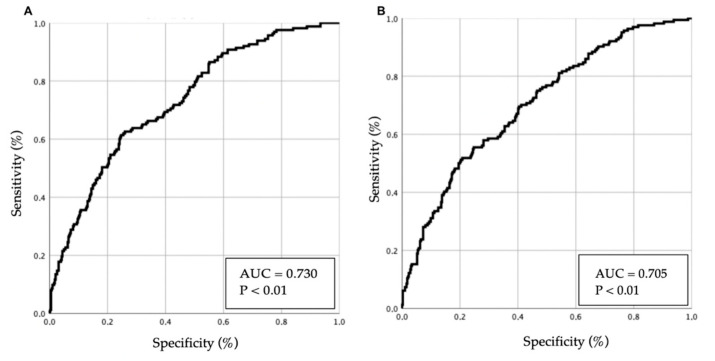
ROC curves of multivariable models for predicting 12-month mortality in patients with acute heart failure. Panels (**A**,**B**) correspond to model 1 and model 2, respectively. The diagonal line represents chance (AUC = 0.5); ROC curves illustrate each model’s discriminative ability.

Two multivariable models were developed using different variable-selection methods (conditional backward method and enter method), and their results are presented in Table 4. Predictive performance is illustrated in Figure 3 (ROC curves).

In the 12-month analysis, age and previous episodes of AHF remained significantly associated with mortality, whereas valvular heart disease lost statistical significance. AF as a PF continued to show a trend toward a protective effect, although without reaching statistical significance.

Taken together, these findings highlight both shared and time-specific predictors of mortality. Age, NYHA functional class, and creatinine levels consistently emerged as significant prognostic factors across both short- and long-term follow-up. In contrast, ischemic heart disease, valvular heart disease, and ACS-related AHF were primarily associated with 30-day outcomes, while previous AHF episodes, hemoglobin levels, and post-episode hospital admission were more strongly linked to 12-month mortality.

## 4. Discussion

HF is a prevalent condition and one of the leading causes of hospitalization, with a significant impact on both patient quality of life and healthcare systems. In particular, AHF poses a clinical challenge due to its complexity and frequent need for hospital admission. Understanding the prognostic factors that influence its course is essential to optimize management and improve clinical outcomes, especially in older adults, where frailty and the coexistence of multiple comorbidities shape prognosis and therapeutic response.

Previous studies have shown that the prognostic factors of AHF vary significantly with age. In younger patients, cardiovascular determinants tend to predominate, whereas in older individuals, chronic comorbidities such as DM, ischemic heart disease, and renal disease carry greater prognostic weight [34,35,36,37]. These studies highlight that age not only modifies the prevalence of comorbidities but also influences their clinical impact. For example, Teixeira et al. reported a significantly higher mortality risk in patients aged ≥85 years compared with younger groups (Hazard Ratio [HR] 3.39; 95% CI 1.98–5.83), with a progressive increase in mortality across the ‘middle-old’, ‘old-old’, and ‘oldest-old’ categories in patients hospitalized for AHF [35]. Consistently, Tromp et al. demonstrated markedly higher mortality in patients aged ≥85 years compared with those aged ≤55 years (HR 6.9; 95% CI 4.2–11.4) [37]. In this context, the advanced mean age and high comorbidity burden of our cohort accurately reflect the profile of elderly patients with AHF treated in HED, reinforcing the validity of our findings within the geriatric setting.

Future studies should explore the incorporation of additional variables related to the prior course of HF and the overall comorbidity burden, with the aim of improving prognostic stratification in older patients with AHF.

Our cohort, with a mean age of 80.7 years and a high prevalence of comorbidities such as AF and ischemic heart disease, reflects the clinical profile reported by other hospitals participating in the EAHFE registry, as well as in national registries such as RICA [38,39,40,41]. Gimeno-Miguel et al. identified multimorbidity patterns with prognostic impact in patients with AHF aged ≥85 years, highlighting the cardiovascular pattern, characterized by arrhythmias, valvular disease, and anemia (HR 1.45 in women; 1.60 in men) and the neurovascular pattern, which included dementia (HR 1.62 in women; 1.74 in men) [38]. Complementarily, the Japanese registry by Hamana et al. identified renal impairment (HR 1.17; 95% CI 1.06–1.29) and anemia (HR 0.77; 95% CI 0.67–0.88) as independent predictors of mortality in patients older than 75 years [34]. Taken together, these findings underscore the prognostic relevance of comorbidity burden and analytical abnormalities in elderly patients with AHF.

Age, dementia, and valvular heart disease emerged as consistent predictors across all follow-up periods, reinforcing the importance of comorbidities in risk stratification. In this context, advanced age functions not only as a biological marker but also as a clinical determinant that amplifies the impact of chronic conditions and PFs. Compared with large registries such as ADHERE [42], OPTIMIZE-HF [43], and ESC-HF-LT [44], our cohort included older patients, in whom the prevalence and prognostic impact of comorbidities such as DM, ischemic heart disease, and dementia differ from those observed in younger populations. In our study, DM was not associated with short-term mortality, and only a non-significant trend was observed at 12 months. Similarly, ischemic heart disease showed a clearer association with 30-day mortality but lost prognostic significance at one year. These findings are particularly relevant from a geriatric perspective, as age modifies the clinical expression of disease, alters therapeutic tolerance, and requires a multidimensional approach that integrates functional, cognitive, and social aspects. Age also influences the expression and prognostic weight of these comorbidities, contributing to distinct clinical trajectories in older patients with AHF. Age itself influences the expression and impact of these conditions, contributing to distinct clinical trajectories in older patients with AHF. Other comorbidities, such as a history of HF or prior hospitalization, showed prognostic relevance primarily in long-term follow-up, underscoring the need to differentiate the temporal impact of each variable.

Regarding clinical status at HED presentation, the baseline NYHA functional class, reflecting the patient’s chronic functional status prior to the acute episode, was a key prognostic determinant: class IV was associated with higher mortality at both 30 days and 1 year, whereas class III was linked only to long-term mortality. Hypoxemia and low-output symptoms at admission were associated with poorer 30-day outcomes but lost prognostic significance at 1 year. Similarly, the hypotensive form of AHF showed excess early mortality without long-term impact, consistent with studies on perfusion and cardiogenic shock [45,46]. This pattern may partly reflect a survivor effect, with early deaths occurring predominantly among the most severe acute presentations. These findings underscore the importance of considering the broader clinical context of elderly patients with HF, in whom acute episodes represent only one manifestation of a chronic and evolving disease process. This process is associated with greater baseline clinical vulnerability, as reflected by the poorer long-term prognosis of patients with decompensated chronic HF compared with de novo cases [47]. In this setting, data from Cooper et al. [48] show that the severity of acute symptoms, particularly the degree of congestion at admission, is independently associated with worse one-year outcomes, indicating that these acute episodes constitute critical junctures within a chronic and progressive condition.

This temporal pattern suggests that classical signs of acute decompensation primarily reflect immediate physiological instability and influence early outcomes, whereas long-term prognosis is more strongly determined by chronic disease burden and the overall vulnerability of older patients. In this regard, these findings question the uniform prognostic utility of some of these parameters when incorporated indiscriminately into standardized risk scores applied to geriatric populations, and highlight the importance of considering the time horizon in risk assessment.

PFs were identified in nearly 70% of patients, underscoring their high frequency and clinical relevance. Although some studies report that AF is associated with a similar or worse prognosis in HF [13,49,50], our data suggest a different pattern for rapid AF at admission, showing a non-significant trend toward better long-term survival. This finding is directionally consistent with evidence from large cohorts in which AF identified as a PF is consistently associated with better short-term prognosis (HR 0.67) [51] and confers a significant 90-day survival advantage (HR 0.56) [52], potentially identifying a distinct clinical phenotype. The consistency of this observation across different cohorts suggests that rapid AF in the acute setting may reflect a more favorable clinical phenotype or a better therapeutic response. An alternative explanation may be residual confounding related to baseline frailty or underlying disease severity.

Conversely, ACS was associated with higher mortality, showing a marked short-term trend and a statistically significant association in the long-term multivariable models. This finding is consistent with the available evidence, which quantifies a significant increase in short-term mortality risk when ACS precipitates AHF (HR 1.69) [52], and with data showing an approximately two-fold increase in adjusted one-year mortality (HR ~2.0) [53]. It is also aligned with its recognition as a predictor in validated short-term risk-stratification tools for AHF [54], as well as with the established consensus describing the consistently worse prognosis of HF complicating ACS [55]. These findings confirm that PFs not only trigger decompensation but also exert differentiated prognostic influence depending on the follow-up period. In our cohort, infection as a PF showed a non-significant trend toward higher mortality. This direction of effect is consistent with large-scale studies reporting a significant increase in mortality risk for AHF precipitated by infection (HR 1.51 [52]; HR 1.587 [56]). Other PFs, such as atrial flutter, anemia, hypertensive crisis, or poor therapeutic adherence, were not significantly associated with mortality in our analysis. Although anemia is a recognized adverse prognostic factor in AHF, being independently associated with an increased risk of one-year mortality (HR 1.30 [57] and 1.58 [58]), and non-adherence is associated with more than a two-fold increase in the risk of adverse events (HR 2.07) [59], the lack of association in our study may be explained by sample size or by specific characteristics of the population.

Among the biomarkers, creatinine emerged as a robust predictor across all follow-up intervals, underscoring the role of renal function in the progression of AHF. This finding is supported by meta-analytic evidence demonstrating its independent association with mortality (HR 2.31) in HF [60]. In contrast, in our adjusted models, higher hemoglobin levels were associated with lower long-term mortality. This result is consistent with previous evidence identifying anemia as an adverse prognostic factor [61], as well as studies quantifying a benefit for each unit increase in hemoglobin (HR 0.77) [34]. In older patients, early identification and management of these biomarkers are particularly relevant, as they allow for tailored interventions aligned with physiological capacity and help reduce iatrogenesis from intensive treatments.

These findings reinforce the need to incorporate both comorbidities and PFs into the initial assessment. Current risk-stratification models do not routinely include PFs as prognostic variables [62,63,64]. Our data, although not conclusive, suggest that specific PFs, such as rapid AF and ACS, may contribute to risk stratification, as they showed signals of risk in our analyses, with trends toward different short- and long-term outcomes. This supports the need for further investigation in larger cohorts. In elderly populations, a stratification model that integrates age, comorbidity, frailty, and PFs may enhance clinical decision-making, optimize hospital admissions, and guide interventions more proportionally to functional status and overall prognosis. Including these parameters alongside clinical variables may improve identification of high-risk patients and support more individualized management, enabling tailored therapies and follow-up strategies adapted to each patient’s clinical profile.

In conclusion, our results show that the prognostic impact of risk factors in AHF varies according to the temporal horizon, and that PFs play a relevant role that has often been underestimated. In elderly patients, this temporal approach is particularly important, as it allows for differentiation between determinants influencing the acute phase and those shaping chronic evolution. This framework adds value to risk stratification by identifying which patients require more intensive interventions during the acute phase and which need closer long-term follow-up. Future studies should focus on developing predictive models and therapeutic strategies specifically tailored to the geriatric population with AHF, integrating clinical, biological, and functional factors to support truly personalized care.

### Study Limitations

This study presents several limitations that should be considered when interpreting the results. First, as it was conducted in a single hospital, extrapolation of the findings to other centers should be made with caution, given potential differences in emergency and inpatient care protocols. Second, it should be noted that, since the study is based on the EAHFE registry, it includes only patients treated in the HED, which may limit the comparability of our results with other registries, such as RICA, that are oriented toward patients hospitalized in Internal Medicine units. Furthermore, as this is a secondary analysis of a previously designed registry, the inclusion of variables was restricted to those defined in the original protocol.

In addition, the long follow-up period resulted in a substantial proportion of missing data for certain variables, particularly analytical parameters such as NT-proBNP and troponin, which were not available at our hospital during the initial years of data collection. The absence of these biomarkers may have affected the overall validity and accuracy of the predictive models. Similarly, it was not possible to include echocardiographic parameters, as echocardiography is not routinely performed in the HED. This limits our ability to characterize HF phenotypes and to adjust for systolic function, a key prognostic variable available in hospital-based studies such as that of Ichihara et al. [26]. The absence of this information may affect the comparability of our findings on PFs with studies that are able to incorporate these parameters. Some variables showed wide CI, reflecting lower precision in the estimates due to the small number of events in certain categories, but were retained because of their clinical relevance and potential prognostic value.

Nevertheless, conducting the study in a single HED provided sample homogeneity and reduced variability in data collection and clinical management. Furthermore, since it was carried out in the emergency setting—the main gateway to the healthcare system—it yielded a broad and representative sample that strengthens the robustness of the findings and enables early identification of prognostic factors, which is essential for improving risk stratification and facilitating more efficient care transitions.

## 5. Conclusions

This study identified the main prognostic determinants of mortality in elderly patients hospitalized for AHF, emphasizing the added value of analyzing risk factors across distinct temporal horizons. Well-established predictors such as advanced age, valvular heart disease, and dementia consistently influenced outcomes throughout all follow-up periods. Other variables, including low-output symptoms, history of chronic HF, and baseline NYHA functional class, demonstrated prognostic relevance that varied depending on the time frame analyzed.

Importantly, PFs showed differentiated prognostic impact: ACS was associated with increased short-term mortality, whereas rapid AF emerged as a favorable marker during long-term follow-up.

These findings underscore the importance of incorporating the temporal dimension of prognostic determinants and integrating PFs into risk stratification models. Their inclusion in clinical decision-making may enhance the selection of candidates for intensive therapies, optimize hospitalization strategies, and strengthen continuity of care and long-term outcomes in older patients with AHF. From a geriatric perspective, it is essential to advance toward truly personalized management strategies that consider not only clinical parameters but also the functional, cognitive, and social status of each patient. This multidimensional approach may allow for tailored interventions, reduce unnecessary therapeutic burden, and improve quality of life in a highly vulnerable population.

## Figures and Tables

**Table 1:** Sociodemographic characteristics and clinical profile of patients with AHF included in the study.

	Patients (*n* = 548)
Epidemiological and baseline functional data	
Age (years)	80.7 ± 9.9
Female sex	272 (49.6%)
NYHA Functional Class	
Class I	130 (23.7%)
Class II	241 (44.0%)
Class III	162 (29.6%)
Class IV	15 (2.7%)
Cardiovascular history	
Atrial fibrillation	196 (35.8%)
Ischemic heart disease	130 (23.7%)
Valvular heart disease	108 (19.7%)
Previous episodes of AHF	343 (62.6%)
Non-cardiovascular comorbidities	
COPD	70 (12.8%)
Dementia	31 (5.7%)

AHF, acute heart failure; COPD: chronic obstructive pulmonary disease; NYHA, New York Heart Association. Values are expressed as *n* (%), mean ± standard deviation.

**Table 2:** Clinical characteristics and management of the AHF episode in patients included in the study.

	Patients (*n* = 548)
Clinical status of the patient	
Respiratory rate > 25 breaths/min	37 (6.8%)
Oxygen saturation (%)	93.6 ± 6.7
Congestive signs	428 (78.1%)
Low-output symptoms	14 (2.6%)
Acute heart failure profile	
Hemodynamic profile	
Warm and wet	474 (86.5%)
Cold and wet	64 (11.7%)
Cold and dry	5 (0.9%)
Warm and dry	5 (0.9%)
Precipitating factor	
Infection	190 (34.7%)
Rapid atrial fibrillation	95 (17.3%)
Acute coronary syndrome	31 (5.7%)
Laboratory test results	
Hemoglobin (g/dL)	12.4 ± 5.3
Creatinine (mg/dL)	1.4 ± 1.1
Potassium (mEq/L)	4.5 ± 1.7
Emergency department management	
Hospital admission	421 (76.8%)

Congestive and low-output clinical signs and symptoms considered in the analysis are detailed in Supplementary Table S1. Values are expressed as *n* (%), mean ± standard deviation, or median [interquartile range].

**Table 3:** Comparative summary of multivariable models for predicting 30-day mortality across different clinical scenarios.

	OR	95% CI	*p*	AUC (%)
Model 1				85.8%
Age	1.12	1.07–1.17	<0.01	
Ischemic heart disease	3.07	1.49–6.31	<0.01	
Valvular heart disease	2.16	1.01–4.61	0.05	
Previous HF	1.42	0.64–3.14	0.39	
AHF Types				
Hypertensive (reference category)	-	-	<0.01	
Normotensive	4.52	1.02–20.06	0.05	
Hypotensive without shock	8.28	1.75–39.24	<0.01	
Hypotensive with cardiogenic shock	22.73	1.13–457.66	0.04	
AHF associated with ACS	231.65	27.52–1949.61	<0.01	
NYHA functional class				
Class I (reference category)	-	-	<0.01	
Class II	0.68	0.24–1.94	0.47	
Class III	2.21	0.83–5.87	0.11	
Class IV	16.40	3.52–76.36	<0.01	
Creatinine	1.61	1.27–2.04	<0.01	
Model 2				85.0%
Age	1.12	1.07–1.17	<0.01	
Ischemic heart disease	2.93	1.44–5.98	<0.01	
Acute Heart Failure Types	4.53	1.02–20.17	0.05	
Hypertensive (reference category)	-	-	<0.01	
Normotensive	3.80	0.89–16.16	0.07	
Hypotensive without shock	7.81	1.71–35.71	<0.01	
Hypotensive with cardiogenic shock	15.99	0.83–307.04	0.07	
AHF associated with ACS	187.05	23.66–1478.48	<0.01	
NYHA functional class				
Class I (reference category)	-	-	<0.01	
Class II	0.79	0.29–2.16	0.65	
Class III	2.25	0.86–5.88	0.10	
Class IV	17.59	3.97–77.89	<0.01	
Creatinine	1.60	1.27–2.03	<0.01	

ACS, acute coronary syndrome; AHF, acute heart failure; AUC, area under the curve; CI, confidence interval; HF, heart failure; NYHA, New York Heart Association functional class; OR, odds ratio. AUC values are expressed as (%).

**Table 4:** Comparative summary of multivariable models for predicting 12-month mortality across different clinical scenarios.

	OR	95% CI	*p*	AUC (%)
Model 1				73.0%
Age	1.05	1.02–1.07	<0.01	
Valvular heart disease	1.48	0.91–2.41	0.11	
Previous HF	1.71	1.08–2.73	0.02	
NYHA functional class				
Class I (reference category)	-	-	<0.01	
Class II	1.29	0.71–2.33	0.40	
Class III	2.32	1.28–4.21	<0.01	
Class IV	4.03	1.19–13.68	0.02	
AF as PF	0.77	0.43–1.37	0.37	
ACS as PF	2.44	1.06–5.64	0.04	
Creatinine	1.25	1.04–1.50	0.02	
Hemoglobin	0.88	0.79–0.98	0.02	
Potassium	1.26	0.96–1.65	0.09	
Hospital admission	2.00	1.19–3.36	<0.01	
Model 2				70.5%
Age	1.04	1.02–1.07	<0.01	
Previous HF	1.81	1.16–2.82	<0.01	
NYHA functional class				
Class I (reference category)	-	-	<0.01	
Class II	1.34	0.75–2.39	0.32	
Class III	2.35	1.31–4.19	<0.01	
Class IV	4.55	1.36–15.14	0.01	
Creatinine	1.34	1.12–1.60	<0.01	
Hospital admission	1.97	1.18–3.28	<0.01	

ACS, acute coronary syndrome; AF, atrial fibrillation; AUC, area under the curve; CI, confidence interval; HF, heart failure; NYHA, New York Heart Association functional class; OR, odds ratio; PF, precipitating factor. AUC values are expressed as (%).

## Data Availability

The data supporting the findings of this study are not publicly available due to confidentiality and ethical restrictions. The information originates from the EAHFE registry and access is limited in accordance with current data protection regulations and the ethical agreements established by the relevant research committees.

## Supplementary Materials

The following supporting information can be downloaded at https://www.mdpi.com/article/10.3390/geriatrics11010021/s1, Table S1: Overview of congestive and low-output clinical signs and symptoms included in the analysis; Table S2: Baseline characteristics stratified by 30-day and 12-month mortality; Table S3: Univariate logistic regression analysis of risk factors for 30-day mortality; Table S4: Univariate logistic regression analysis of risk factors for 12-month mortality.

## Abbreviations

The following abbreviations are used in this manuscript:

ACS	Acute Coronary Syndrome
AF	Atrial Fibrillation
AHF	Acute Heart Failure
AUC	Area Under the Curve
CI	Confidence Interval
COPD	Chronic Obstructive Pulmonary Disease
DM	Diabetes Mellitus
EAHFE	Epidemiology of Acute Heart Failure in Emergency Departments
ECG	Electrocardiogram
HF	Heart Failure
HR	Hazard Ratio
HED	Hospital Emergency Department
HUMV	Marqués de Valdecilla University Hospital
IQR	Interquartile Range
MRN	Medical Record Number
NYHA	New York Heart Association
NTproBNP	N-terminal pro-B-type Natriuretic Peptide
OR	Odds Ratio
PF(s)	Precipitating Factor(s)
RICA	National Heart Failure Registry (Registro Nacional de Insuficiencia Cardiaca)
ROC	Receiver Operating Characteristic
SPSS	Statistical Package for the Social Sciences
STEMI	ST-segment elevation myocardial infarction
