# Supplementary material for: Temporal Prognostic Factors in Elderly Patients with Acute Heart Failure: A Cohort Study from a Spanish Emergency Department
Source: Geriatrics (Basel). 2026 Feb 18;11(1):21. doi: 10.3390/geriatrics11010021 (PMC12940665; doi:10.3390/geriatrics11010021)
Supplement: Supplementary file 1 [file geriatrics-11-00021-s001.zip › Supplementary Table 1. Overview of congestive and low output clinical signs and symptoms included in the analysis..pdf]

Supplementary Table S1. Overview of congestive and low-output clinical signs and symptoms included in the analysis.

Congestive Symptoms and Signs
Dyspnea
Orthopnea
Paroxysmal nocturnal dyspnea
Elevated jugular venous pressure
Hepatomegaly
Peripheral edema
Resting tachycardia
Third heart sound (S3)
Pulmonary crackles
Cardiomegaly
Pleural effusion
Low-output signs
Distal coldness
Skin pallor
Delayed capillary refill
Livedo reticularis
Altered mental status
