# Supplementary material for: Temporal Prognostic Factors in Elderly Patients with Acute Heart Failure: A Cohort Study from a Spanish Emergency Department
Source: Geriatrics (Basel). 2026 Feb 18;11(1):21. doi: 10.3390/geriatrics11010021 (PMC12940665; doi:10.3390/geriatrics11010021)
Supplement: Supplementary file 1 [file geriatrics-11-00021-s001.zip › Supplementary Table 2. Baseline characteristics of the study population stratified by 30-day and 12-month mortality..pdf]

Supplementary Table 2. Baseline characteristics of the study population stratified by 30-day and 12-month mortality

	30-Day mortality		12-Month Mortality	
	**Yes**	**No**	**Yes**	**No**
Epidemiological and baseline functional data				
Age	85.9 ± 7.3	80.2 ± 10.0	83.5 ± 9.0	79.5 ± 10.0
Sex				
Female	33 (12.1)	239 (87.9)	81 (29.8)	191 (70.2)
Male	20 (7.2)	256 (92.8)	83 (30.1)	193 (69.9)
NYHA Functional Class				
Class I (reference category)	9 (6.9)	121 (93.1)	24 (18.5)	106 (81.5)
Class II	15 (6.2)	226 (93.8)	67 (27.8)	174 (72.2)
Class III	22 (13.6)	140 (86.4)	64 (39.5)	98 (60.5)
Class IV	7 (46.7)	8 (53.5)	9 (60.0)	6 (40.0)
Comorbid Conditions				
Diabetes Mellitus				
Yes	22 (11.4)	171 (88.6)	68 (35.2)	125 (64.8)
No	31 (8.7)	324 (91.3)	96 (27.0)	259 (73.0)
Ischemic heart disease				
Yes	20 (15.4)	110 (84.6)	47 (36.2)	83 (63.8)
No	33 (7.9)	385 (92.1)	117 (28.0)	301 (72.0)
Atrial fibrillation				
Yes	15 (7.7)	181 (92.3)	52 (26.5)	144 (73.5)
No	38 (10.8)	314 (89.2)	112 (31.8)	240 (68.2)
Atrial flutter				
Yes	2 (20.0)	8 (80.0)	3 (30.0)	7 (70.0)
No	51 (9.5)	487 (90.5)	161 (29.9)	377 (70.1)
Valvular heart disease				
Yes	17 (15.7)	91 (84.3)	42 (38.9)	66 (61.1))
No	36 (8.2)	404 (91.8)	122 (27.7)	318 (72.3)
Previous episodes of AHF				
Yes	38 (11.1)	305 (88.9)	121 (35.3)	222 (64.7)
No	15 (7.3)	190 (92.7)	43 (21.0)	162 (79.0)
COPD				
Yes	5 (7.1)	65 (92.9)	25 (35.7)	45 (64.3)
No	48 (10.0)	430 (90.0)	139 (29.1)	339 (70.9)
Dementia				
Yes	7 (22.6)	24 (77.4)	17 (54.8)	14 (45.2)
No	46 (8.9)	471 (91.1)	147 (28.4)	370 (71.6)
Clinical status of the patient				
Respiratory rate				
< 25 breaths/min	121 (26.7)	333 (73.3)	170 (37.4)	284 (62.6)
25 – 29 breaths/min	22 (38.6)	35 (61.4)	27 (47.4)	30 (52.6)
> 30 breaths/min	12 (32.4)	25 (67.6)	19 (51.4)	18 (48.6)
Oxygen saturation	89.2 ± 9.1	94.0 ± 6.3	93.1 ± 6.1	93.8 ± 6.9

Supplementary Table 2 (continued).

Low-output symptoms				
Yes	4 (28.6)	10 (71.4)	7 (50.0)	7 (50.0)
No	53 (9.9)	481 (90.1)	155 (29.5)	371 (70.5)
Lower limb edema				
Yes	35 (9.8)	323 (90.2)	110 (30.7)	248 (69.3)
No	18 (9.5)	172 (90.5)	54 (28.4)	136 (71.6)
Pulmonary crackles				
Yes	35 (9.5)	332 (90.5)	109 (29.7)	258 (70.3)
No	18 (9.9)	163 (90.1)	55 (30.4)	126 (69.6)
Acute heart failure profile				
Precipitating factor				
Infection				
Yes	22 (11.6)	168 (88.4)	58 (30.5)	132 (69.5)
No	31 (8.7)	327 (91.3)	106 (29.6)	252 (70.4)
Rapid atrial fibrillation				
Yes	6 (6.3)	89 (93.7)	20 (21.1)	75 (78.9)
No	47 (10.4)	406 (89.6)	144 (31.8)	309 (68.2)
Atrial flutter				
Yes	1 (16.7)	5 (83.3)	3 (50.0)	3 (50.0)
No	52 (9.6)	490 (90.4)	161 (29.7)	381 (70.3)
Anemia				
Yes	3 (9.4)	29 (90.6)	12 (37.5)	20 (62.5)
No	50 (9,7)	466 (90.3)	152 (29.5)	364 (70.5)
Hypertensive crisis				
Yes	1 (5.6)	17 (94.4)	4 (22.2)	14 (77.8)
No	52 (9.8)	478 (90.2)	160 (30.2)	370 (69.8)
Acute coronary síndrome (ACS)				
Yes	6 (19.4)	25 (80.6)	14 (45.2)	17 (54.8)
No	47 (9.1)	470 (90.9)	150 (29.0)	367 (71.0)
Therapeutic non-adherence				
Yes	0 (0.0)	26 (100)	7 (26.9)	19 (73.1)
No	53 (10.2)	469 (89.8)	157 (30.1)	365 (69.9)
Acute Heart Failure Types				
Hypertensive (reference category)	3 (3.4)	86 (96.6)	20 (22.5)	69 (77.5)
Normotensive	29 (7.9)	337 (92.1)	106 (29.0)	260 (71.0)
Hypotensive without shock	14 (18.7)	61 (81.3)	29 (38.7)	46 (61.3)
Hypotensive with cardiogenic shock	2 (33.3)	4 (66.7)	3 (50.0)	3 (50.0)
Acute heart failure associated with ACS	7 (58.3)	5 (41.7)	6 (50.0)	6 (50.0)
Complementary tests				
ECG detected LVH				
Yes	1 (3.2)	30 (96.8)	4 (12.9)	27 (87.1)
No	52 (10.1)	465 (89.9)	160 (30.9)	357 (69.1)
Hemoglobin (gr/dL)	11.8 $\pm$ 1.8	12.5 $\pm$ 5.6	11.7 $\pm$ 1.9	12.7 $\pm$ 6.1
Creatinine	2.0 $\pm$ 1.7	1.3 $\pm$1.0	1.6 $\pm$ 1.3	1.3 $\pm$ 0.9
Potassium (mEq/L)	4.6 $\pm$ 0.9	4.3 $\pm$ 1.7	4.67$\pm$ 2.8	4.3 $\pm$ 0.7
NTproBNP (pmol/L)	31744.4 $\pm$48542.5	7217.2 $\pm$9646.3	21683.1 $\pm$34910.7	6198.6 $\pm$7466.1

Troponin	20.6 ± 79.4	14.0 ± 90.3	25.6 ± 135.6	10.6 ± 64.0

Supplementary Table 2 (continued).

Emergency department management				
IMV				
Yes	2 (20.0)	8 (80.0)	3 (30.0)	7 (70.0)
No	51 (9.5)	487 (90.5)	161 (29.9)	377 (70.1)
Intravenous nitroglycerin				
Yes	4 (16.0)	21 (84.0)	10 (40.0)	15 (60.0)
No	49 (9,4)	474 (90.6)	154 (29.4)	369 (70.6)
Hospital admission				
Yes	46 (10.9)	375 (89.1)	140 (33.3)	281 (66.7)
No	7 (5.5)	120 (94.5)	24 (18.9)	103 (81.1)

ACS: Acute heart failure associated with acute coronary syndrome; AHF, acute heart failure; COPD: chronic obstructive pulmonary disease; ECG: Electrocardiogram; IMV: Invasive mechanical ventilation; LVH: Left ventricular hypertrophy; NYHA, New York Heart Association.

Values are expressed as *n* (%), mean ± standard deviation, or median [interquartile range].
